# Phenolic Profile, Antioxidant and DNA-Protective Capacity, and Microscopic Characters of *Ailanthus altissima* Aerial Substances

**DOI:** 10.3390/plants12040920

**Published:** 2023-02-17

**Authors:** Tsvetelina Andonova, Yordan Muhovski, Iliya Slavov, Radka Vrancheva, Vasil Georgiev, Elena Apostolova, Samir Naimov, Rumen Mladenov, Atanas Pavlov, Ivanka Dimitrova-Dyulgerova

**Affiliations:** 1 Department of Botany and Biological Education, Faculty of Biology, University of Plovdiv “Paisii Hilendarski”, 4000 Plovdiv, Bulgaria; 2 Life Sciences Department, Walloon Agricultural Research Centre, 5030 Gembloux, Belgium; 3 Department of Biology, Faculty of Pharmacy, Medical University of Varna, 9000 Varna, Bulgaria; 4 Department of Analytical Chemistry and Physical Chemistry, Technological Faculty, University of Food Technologies, 4002 Plovdiv, Bulgaria; 5 Laboratory of Cell Biosystems, Institute of Microbiology, Bulgarian Academy of Sciences, 139 Ruski Blvd., 4000 Plovdiv, Bulgaria; 6 Department of Plant Physiology and Molecular Biology, Faculty of Biology, University of Plovdiv “Paisii Hilendarski”, 4000 Plovdiv, Bulgaria; 7 Department of Medical Biochemistry, Faculty of Pharmacy, Medical University of Plovdiv, 15A Vasil Aprilov Blvd., 4002 Plovdiv, Bulgaria

**Keywords:** *Ailanthus altissima*, antioxidant activity, DNA protection, flavonoids, phenolic acids, microscopic diagnostic characters

## Abstract

Invasive species as sources of natural components are of increasing interest for scientific research. This is the case of *Ailanthus altissima*, which belongs to the top 100 of the most dangerous invasive plant species in Europe, and which is the subject of the present study. The purpose of the research was to analyze the main phenolic compounds in the flowers, leaves, and stem bark of *A. altissima* and determine the DNA-protective and antioxidant potential of their ethanolic extracts. HPLC profiling revealed the presence of 6 flavonoids and 10 phenolic acids, of which 15 were found in flowers, 14 in leaves, and 11 in the stem bark. Rutin (5.68 mg/g dw in flowers), hesperidin (2.67 mg/g dw in leaves) and (+)-catechin (2.15 mg/g dw in stem bark) were the best-represented flavonoids. Rosmarinic (10.32 mg/g dw in leaves) and salicylic (6.19 mg/g dw in leaves) acids were predominant among phenolic acids. All plant extracts tested showed in vitro antioxidant activity (determined by DPPH, ABTS, FRAP, and CUPRAC assays) and DNA-protection capacity (assay with supercoiled plasmid DNA—pUC19). The highest antioxidant activity was recorded in the flower parts (in the range from 661 to 893 mmol TE/g dw), followed by the leaves. A DNA protective potential for *A*. *altissima* leaf and flower extracts has not been established to date. In addition, the main microscopic diagnostic features of studied plant substances were described, with data for the flower parts being reported for the first time. The present study proves that *A. altissima* could be a natural source of DNA protection and antioxidants.

## 1. Introduction

In the search for new sources of bioactive compounds for the prevention and treatment of various diseases, there is a growing scientific interest in widespread species that are invasive to a number of countries. By suppressing and displacing native species they pose a threat to biodiversity, but at the same time they can be a valuable and accessible natural resource of compounds exhibiting a range of pharmacological effects [1,2,3,4,5]. Extensive studies on the chemical composition of such species, as well as on manifested biological effects of plant extracts from them, enrich the set of plant species, and sources of medicinal raw materials. The object of the present study is a tree species that is widespread and alien to the territory of Bulgaria and Europe, namely *Ailanthus altissima* (Mill.) Swingle (ailanthus, tree of heaven), Simaroubaceae. Its origin is associated with Southeast Asia, and more than a century ago it was imported into the country for decorative purposes, but it became wild and quickly spread. The species is currently classified as highly invasive and belongs to the top 10 dangerous species in Bulgaria and the top 100 for Europe [1]. 

At the same time, however, data on the presence of some valuable secondary metabolites (simple phenols, flavonoids, phenolic acids, lignans, coumarins, polyphenols, steroids, terpenes and terpenoids, alkaloids, volatile components, etc.) are reported for *A. altissima*, which are the basis of its rich pharmacological potential (antioxidant, antimicrobial, antiviral, phytotoxic, anti-inflammatory, anti-parasitic, anti-tumor, and anti-insecticide properties, etc.) [2,4,5,6,7,8,9,10,11,12,13,14,15,16]. Additionally, the species finds application as a traditional remedy [4,5,6,8,17]. The widespread distribution of *A. altissima* in Bulgaria, as well as in a number of other countries on the territory of Europe and other continents, turning it into a cheap and accessible natural resource [2,3,5]. 

In the fruits, leaves, and stem bark, simple phenolic compounds have been found: phenol, resorcinol, pyrogallol, hydroquinone, arbutin, vanillin, etc. [12,13,18,19,20,21]. With the help of various analytical techniques in the plant parts of *A. altissima,* some representatives of phenolic acids and their derivatives have been identified: benzoic acid, ferulic acid, gallic acid; vanillic acid, caffeic acid, etc. poliphenols [8,9,10,14,19,20,21,22,23,24,25,26,27,28,29,30,31]. According to Poljuha et al. [27], caffeic acid and chlorogenic acid, are predominant in the composition of extracts from dried and fresh leaves of A. *altissima*. The second, the authors suggest, is involved in the protection of the plant, it has also been proven in other plant substances of the species [9,14,23,26]. The increased scientific interest in phenolic acids in the species also reveals the presence of ellagic acid, syringic acid, salicylic acid, *p*-coumaric acid, protocatechuic acid, *p*-hydroxybenzoic acid, etc. [10,14,19,21,23,24,25,26,28,30]. Along with phenolic acids, flavonoids are a group of phenolic metabolites of great pharmacological importance and the focus of part of scientific research on the species. Various flavonoid aglycones have been found in the extracts of the above-ground plant parts: apigenin, luteolin, kaempferol, quercetin, rutin, hyperoside, as well as their glycosides [13,14,19,21,23,24,26,27,28,29,30,32]. The catechins are established: catechin, epicatechin [9,21,26], gallocatechin, epicatechinalate, procyanidins (B1, B2, B3, B4), taxifolin and myricitrin [21]. Resveratrol, for which multiple pharmacological effects are known, has been found in the leaves and flowers of tree species [21,26]. The presence of these two groups of phenols, which are proven antioxidants, suggests the possibility of such activity in the plant species that is the subject of the present work. Various scientific publications report on the antioxidant capacity of leaf extracts of *A. altissima*. Rahman et al. [11] demonstrated a strong ability of the ethyl acetate leaf fraction to scavenge free radicals, stronger even than the synthetic antioxidant butylated hydroxyanisole. Studies by Albouchi et al. [9] proved a strong dose-dependent antioxidant activity for the methanol leaf extract and its hydro-distilled residues. Luís et al. [23] found a positive linear correlation between the antioxidant power of crude (ethanol, methanol, acetone, and water-alcohol 50:50) extracts (from stems, stalks, and leaves) of the tree species and the total phenolic content. High free radical reducing power of n-butanol and ethyl acetate leaf fractions was reported [17]. Comparative analyzes (ABTS, DPPH, and FRAP) demonstrate differences in the values of the measured activities, depending on the state of the leaf plant substances (fresh, dry, whole, and fragmented) and the type of solvent used (methanol, water) [27]. Ethanol leaf extracts, obtained by two different methods—reflux or ultrasound—also show good ABTS and DPPH results, correlating with the amounts of phenolic compounds in them [13]. Scientific studies report on the antioxidant properties of the carotenoid fraction of the stem bark [33] and the wood aqueous extracts from *A. altissima* [30,34]. High antioxidant activity was reported for bark extracts (ABTS) and lower for leaves (DPPH) from *A. altissima* [14]. Individual isolated compounds (lignans, vanillic acid, ferulic acid derivative, etc.) from root barks [8,35] and seed oil [36] exhibit a powerful ability to scavange free radicals. A significant antioxidant power has been established for the fruits and the possible mechanisms of action related to the regulation of certain signaling pathways have been indicated. Recent studies have reported an increase in the antioxidant potential of the species with the application of nanotechnology, almost equal to that of ascorbic acid [37].

Based on the review of the literature data, it is clear that *A. altissima* is a promising subject for phytochemical and biological studies, and for this reason the need to supplement the data follows. In addition, the species has been poorly researched for the territory of Bulgaria [15,33].

In view of the above, the purpose and tasks of the present study were determined, namely: the study of flowers, leaves, and stem barks of *A. altissima* for phenolic components with HPLC technique, proof of antioxidant and DNA protective potential of ethanol extracts, and determination of main microscopic diagnostic features of the three plant substances.

## 2. Results

### 2.1. Phenolic Profile (HPLC Analysis) of Aerial Plant Substances of A. altissima

In ethanol extracts of the above-ground substances of *A. altissima*, 16 phenolic compounds (6 flavonoids and 10 phenolic acids) were identified by HPLC analysis (Table 1). Among the flavonoids identified and quantified, the highest content in the leaves were hesperidin (2.67 mg/g dw), rutin (1.02 mg/g dw), and (+)-catechin (0.95 mg/g dw). The other two established flavonoids (−)-epicatechin and quercetin were in much lower amounts. In the flowers, the concentration of rutin (5.68 mg/g dw) was the highest, followed by (+)-catechin, but with a 3.7 times lower concentration than that of rutin. The rest of the flavonoids identified in the flowers were in much lower amounts (0.72–0.13 mg/g dw). Kaempferol was absent in the extracts obtained from the flowers and leaves of *A. altissima.* In stem bark, the catechins were best represented: (+)-catechin and (−)-epicatechin, where amounts of 2.15 mg/g dw and 0.54 mg/g dw were reported, respectively. Kaempferol and quercetin were poorly represented, and rutin and hesperidin were missing in this substance (Table 1). Representative HPLC chromatograms of the phenolic compounds of stem bark extracts and the corresponding standards are added in the Supplementary Materials (Figure S1).

Of the ten phenolic acids investigated, in the leaf extracts of *A. altissima*, nine were found, among which rosmarinic acid was in the highest content (10.32 mg/g dw), followed by salicylic acid (6.19 mg/g dw) (Table 1). The same two acids were predominant in the substances of flowers, but in times (1.6 and 5) lower concentrations than those in the leaves, followed by chlorogenic, *p*-coumaric and ferulic acids. The remaining phenolic acids in leaves and flowers from *A. altissima* were in much lower amounts. The lowest content in the leaves was found for caffeic and ferulic (0.13 and 0.11 mg/g dw), and in the flower parts for gallic and protocatechuic (0.16 and 0.15 mg/g dw) acids. Unlike the flowers, protocatechuic acid was absent in the leaves. Seven phenolic acids were identified in the bark extracts, of which vanillic, chlorogenic, and rosmarinic acids were the best represented in the range of 0.7–0.1 mg/g dw. Vanillic acid is in the highest content in barks (4.5–8 times more) compared to flowers and leaves. Protocatechuic, caffeic, and *p*-coumaric acids were absent in stem barks of *A. altissima.*

### 2.2. In Vitro Antioxidant Capacity

The results of the conducted in vitro ABTS, DPPH, CUPRAC and FRAP assays are presented in Table 2. As can be seen from the table, all ethanolic extracts demonstrated antioxidant activity (AOA), but it was most strongly expressed in the flower ones by ABTS (893.14 mmol TE/g dw), CUPRAC (789.54 mmol TE/g dw) and DPPH (729.72 mmol TE/g dw) assays. The leaf extracts showed 1.4–3.2 times lower antioxidant activity, compared with the flower extracts in the same three tests. Better activity for the leaf extracts was established in metal-reducing (FRAP, CUPRAC) assays, compared with radical-scavenging ones (ABTS, DPPH). Antioxidant activity of the stem bark extracts was 28–77 times lower than others (between 10.22 and 31.24 mmol TE/g dw).

### 2.3. In Vitro DNA-Protective Capacity

**Figure 1:**
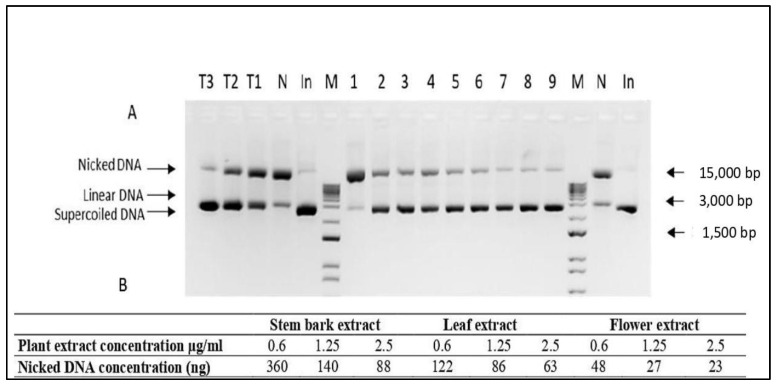
DNA nicking protection assay with (**A**) 1.5% agarose gel electrophoresis, and (**B**) relative concentration of necked plasmid DNA. 1–3—*A. altissima* stem bark extract at concentrations of 0.6, 1.25, and 2.5 μg/mL; 4–6—*A. altissima* leaf extract at concentrations of 0.6, 1.25, and 2.5 μg/mL; 7–9—*A. altissima* flower extract at concentrations of 0.6, 1.25, and 2.5 μg/mL. T3—Trolox 100 μg/mL; T2—Trolox 50 μg/mL; T1—Trolox 25 μg/mL; N—negative control; In-pUC19 input; M-Zip Ruler 2 (Thermo Scientific, SM1373).

In order to determine the DNA protective capacity of leaf, flower, and stem bark extracts of *A. altissima* serial dilutions of each extract were tested in in vitro DNA nicking assay. All extracts were tested in three different concentrations—0.6, 1.25, and 2.5 µg/mL (Figure 1). As expected for all extracts inverse correlation between extract concentration and the amount of nicked DNA was found. The best DNA protective effect was observed when flower extracts were applied, where a four-fold decrease in extract concentration resulted in a two-fold increase in the amount of nicked DNA. Depside the higher total amount of nicked DNA, a similar pattern was observed when leaf extracts were applied as DNA protective agents. The lowest DNA protective activity was found for stem bark extracts, where a four-fold decrease in extract concentration resulted in a four-fold change in DNA concentration. 

### 2.4. Light Microscopy Analysis of Ailanthus altissima Aerial Plant Substances

**Figure 2:**
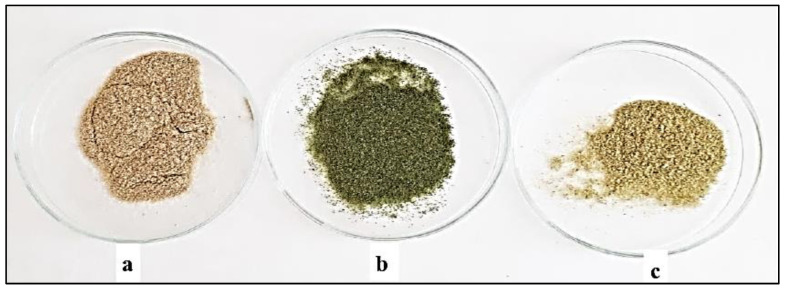
Powdered plant substances from stem bark (**a**), leaves (**b**) and flowers (**c**) from *Ailanthus altissima*.

Powdered stem bark, leaves and flowers of *A. altissima* (Figure 2) were subjected to a microscopic analysis in order to determine the main diagnostic characters. They have an important role in the identification of medicinal plant substances that find pharmaceutical application. Microscopical examination is part of the pharmacognostic analysis in the pharmacopoeial articles.

**Figure 3:**
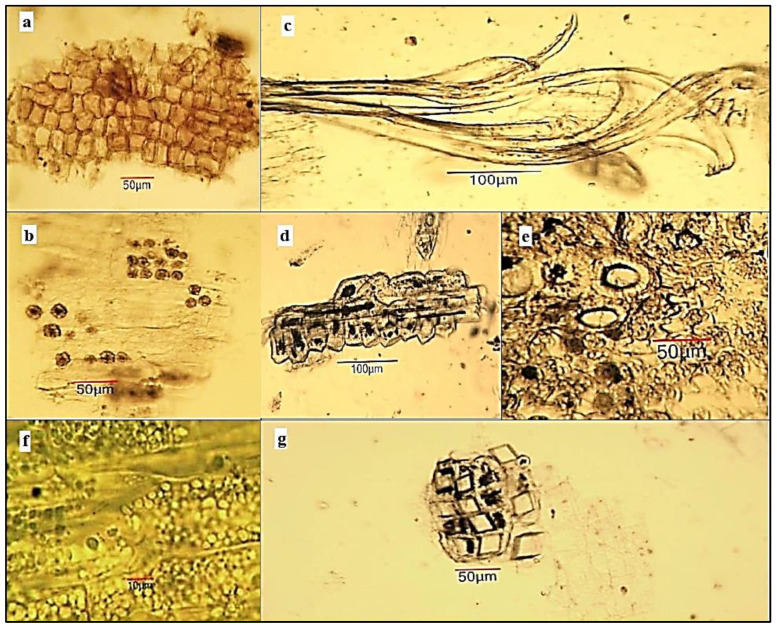
Microphotos of powdered stem bark substance of *Ailanthus altissima*: (**a**) cork on surface view; (**b**) phloem parenchyma with crystalline druses; (**c**) phloem fiber bundle; (**d**) sheath of sclereids around phloem fibers; (**e**) fragment of phloem with schizogenous oil gland; (**f**) fragment of phelloderma with chloroplasts; (**g**) oxalate crystal cubes around a group of sclereids.

*Microscopical examination of powdered stem bark substance of A. altissima.* The powder is light grey (Figure 2a). Examined under a microscope using *chloral hydrate solution*, the powder shows the following diagnostic characters (Figure 3): fragments of brownish cork tissue in front view; fragments of phloem parenchyma with oxalate druses (10–20 μm in diameter); isolated bundles of thick-wall fibers or fragment of fibres, usually associated in groups with sclereids; isolated or groups of sclereids, often accompanied by oxalate crystal prisms; fragments of the phloem containing large secretory schizogenous oil glands (about 50 μm in diameter); fragments of pheloderma; isolated oxalate cluster crystals.

**Figure 4:**
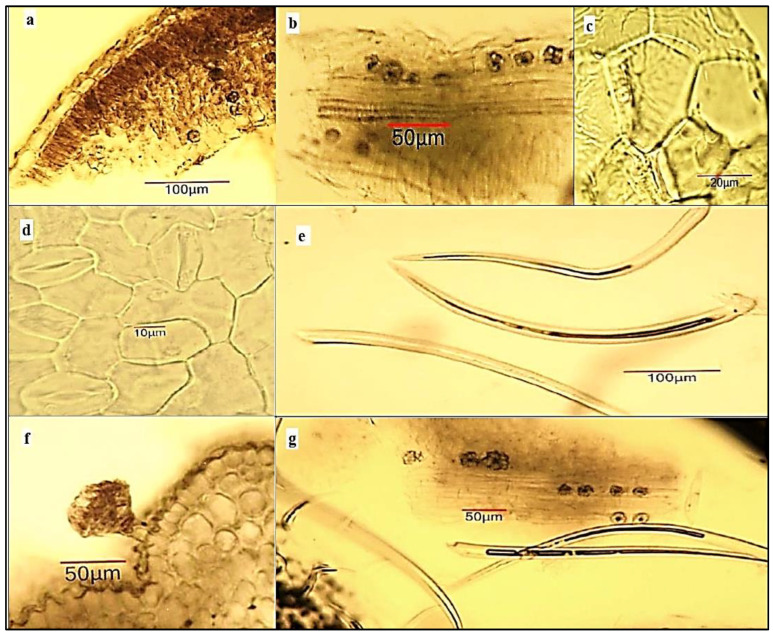
Microphotos of powdered leaf substance of *Ailanthus altissima*: (**a**) fragment of the bifacial leaf lamina in cross section with 1–2 layers of palisade parenchyma, spongy parenchyma and crystalline druses of calcium oxalate; (**b**) oxalate druses around the vascular bundle; (**c**) upper leaf epidermis in surface view; (**d**) lower leaf epidermis in surface view with anomocytic type of stomata; (**e**) isolated unicellular curved covering trichomes; (**f**) glandular head trichome along vein; (**g**) unicellular covering trichomes and fragment of spongy parenchyma with oxalate druses.

*Microscopical examination of powdered leaf substance of A. altissima.* The powder is green (Figure 2b). Examined under a microscope using *chloral hydrate solution*, the powder shows the following diagnostic characters: many fragments of bilateral leaf lamina in cross section containing cells of upper and lower epidermis, palisade and spongy mesophyll, some of which contain oxalate druses; fragments of the upper epidermis in surface view composed of polygonal cells with straight anticlinal walls and striations on the cuticle; fragments of the lower epidermis in anfas composed of cells with wavier walls and anomocytic type of stomata; many covering unicellular trichomes—straight and curved, varying from 50 to 500 μm in length, isolated or with epidermal fragments; glandular trichomes with a multicellular head (about 50 μm in diameter) and a short two-celled stalk (70–80 μm long), located mainly on the leaf veins; fragments of vascular bundle associated with parenchyma and oxalate druses (Figure 4).

**Figure 5:**
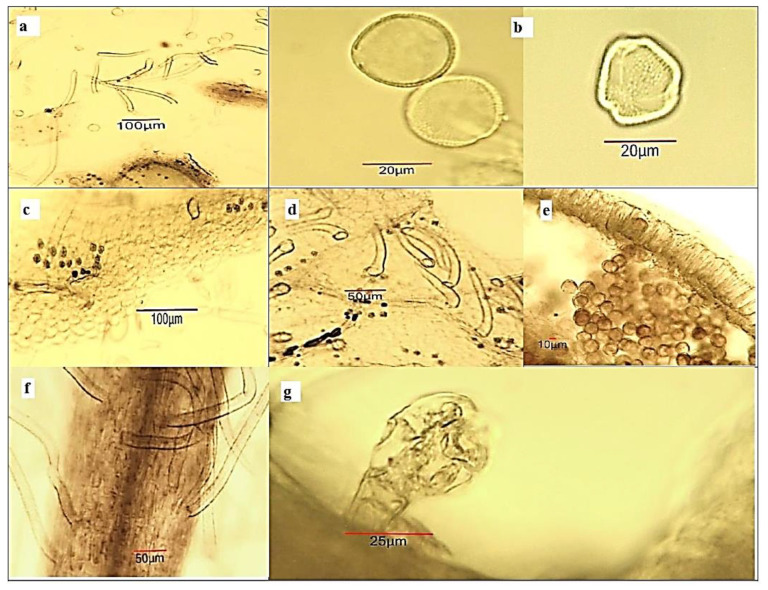
Microphotos of powdered flower substance of *Ailanthus altissima*: (**a**) scattered pollen grains and covering trichomes; (**b**) pollen grains with a pitted exine and 3 pores; (**c**) petal fragment with papillose epidermis and crystal druses, (**d**) sepal fragment with unicellular covering trichomes and crystal druses located mainly around the veins; (**e**) fragment of anther wall with exothecium and endothecium, and pollen grains; (**f**) fragment of funiculus with covering trichomes; (**g**) glandular hair trichome on flower stalk.

*Microscopical examination of powdered flower substance of A. altissima.* The powder is yellow-green (Figure 2c) and shows the following important diagnostic characters in *chloral hydrate solution* (Figure 5): many spherical pollen grains, about 30 µm in diameter, with a pitted exine and three pores; many single-celled linear covering trichomes (short ones—up to 50 μm and long ones—up to 350 μm in length)—isolated and associated with the flower fragments; oxalate druses—isolated and/or included in fragments of the perianthium; fragments of the sepals with a hairy epidermis; fragments of the petals with a papillose epidermis; fragments of the anther with pollen grains; fragments of the funiculus with long covering trichomes in the lower 1/3 to 1/2 part; fragments of the flower stalk with glandular trichomes with a multicellular head and a short two-celled stalk.

## 3. Discussion

The data from the analyzes performed are confirmed by the studies of other authors on the phenolic profile of *A. altissima*. Luís et al. [23] indicated the content of quercetin in acetone and methanol extracts and its absence in ethanol and water extracts. Using chromatographic methods in ethanol leaf extracts, Barakat [29] described nine flavone glucosides, among which kaempferol-3-O-glucoside and quercetin-3-O-glucoside. According to the aforementioned authors, quercetin and rutin are contained in 70% ethanol leaf extract, similar to our data, but the difference is observed regarding the presence of kaempferol established by them, which we found only from the type of stem bark. Quercetin in leaf extracts from *A. altissima*, with solvent methanol was also confirmed by Jin et al. [32], together with other biologically active compounds (astragalin, luteolin, scopolin, scopoletin, etc.).

The polyphenolic leaf profile established by Filippi et al. [21] with the help of the HPLC technique, also confirmed the results established by us, namely: the absence of kaempferol in this plant part; the presence of the flavonoids catechin and epicatechin, and the dominant presence of rutin (many times higher, compared with the quantitative values of the other flavonoids determined by them). The quercetin aglycon was presented in all three of our plant extracts, while the same authors indicated the content of different quercetin glycosides. The similarity of the results obtained by Filippi et al. [21] compared with ours could be explained by the use of the same solvent (ethanol) in obtaining the extracts. The four flavonoids—rutin, quercetin, epicatechin, and catechin have been proven in the leaves and flowers of *A. altissima* in the studies reported by Marinaș et al. [26]. According to their research, the content of rutin (0.59 mg/g dw in leaf and 0.86 mg/g dw in flower) and epicatechin was the highest, and resveratrol (0.01 mg/g dw) was the lowest. Rutin was also confirmed in our research but in significantly higher amounts (1.7 times more in leaves and 6.6 times in flowers). The amounts of catechin measured by us are times higher (24 times for leaves and 32 times for flowers) compared with the data of Marinaș et al. [26]. The similarity in quantitative data was found only for the flavonoid epicatechin. The authors also used ethanol as an extractant to obtain the plant extracts, but using a different methodology (triple extraction with ultrasound). The temperature processing of the plant material (especially above 100 °C) leads to a more complete extraction of the phenolic components as a result of the disintegration of the cellular structures, which could explain the higher amounts found in the study. Quercetin and various phenolic compounds were chromatographically identified in leaf extracts of *A. altissima* also by Al-Hashumi et al. [20]. Comparative HPLC analysis provided by Poljuha et al. [27] regarding the efficiency of extracting phenolic components from fresh and dried leaves showed the presence of two groups of flavonoids in the woody species: flavones (glycosides of apigenin and luteolin) and flavonols (glycosides of kaempferol and quercetin). The phenolic characterization of methanol leaf extracts of ailanthus revealed mainly quercetin derivatives [28]. In contrast to our results, the same authors did not report the presence of gallic and caffeic acids, but identified their derivatives, as well as those of *p*-coumaric, ferulic, hydroxycinnamic, hydroxybenzoic, elagotannins, gallotannins, and other phenolic components. Seven of the identified phenolic acids (gallic, vanillic, caffeic, *p*-coumaric, syringic, ferulic, and chlorogenic acids) were described by Luís et al. [23] as part of the composition of various extracts (ethanol, acetone, methanol, and hydro-alcohol) obtained from leaves and branches. The authors found higher quantitative values of the investigated phenolic components in comparison with our results. The highest difference was observed in the ratio of ferulic (14.21 mg/g dw), chlorogenic and syringic acids, which were dominant in the composition of their leaf extracts, whereas the same components were the least represented (ferulic) in the research sample. Albouchi et al. [9] also proved gallic and chlorogenic acids, as well as the flavonoids epicatechin and rutin in ailanthus leaves, but a comparison could not be made due to the lack of quantitative values. According to Poljuha et al. [27], the amounts of caffeic, chlorogenic, *p*-coumaric, and ferulic acids in dry ground leaves of *A. altissima* were significantly higher compared to fresh, whole, or crushed plant parts. The presence of the acids mentioned by the authors was also confirmed by our research. The chlorogenic, caffeic and syringic acids determined by us were in very close amounts to those reported by Marinaș et al. [26]. The authors also proved a low content of ferulic acid in the leaves among all analyzed phenolic acids. In contrast to Marinaș et al. [26], we did not detect protocatechuic acid in the leaves. In a study by the same authors, *p*-coumaric acid is in a higher content in leaves than in flowers (unlike us). Al-Hashumi et al. [20], through HPLC analysis, proved carboxylic and phenolic acids in the composition of the leaves of the tree species, among which gallic, *p*-hydroxybenzoic, and benzoic acids. Rutin, gallic, ellagic acid, ethyl gallate, and other phenolic components have been isolated and purified from the flowers of the species, based on spectral data and silica gel chromatography, without specifying their amounts for comparison [24]. Gallic acid in a leaf extract was also indicated by Filippi et al. [21], together with ellagic acid, methyl gallate, etc. According to Aissani et al. [30], aqueous extract of the bark and wood of *A. altissima* had the highest presence of quinic and syringic acids, but gallic, protocatechuic, caffeic, *p*-coumaric and trans-frulic acids were also present.

From the above, it can be seen that the studies on the content of phenolic components in the woody species were mainly on its leaf extracts, and single publications report data on their content in its stem barks [30] and in the flowers [24,26]. 

A number of literature sources indicated the presence of the antioxidant potential of *A. altissima.* Luís et al. [23] reported a high level in leaf extracts (methanol, aqueous, hydro-ethanol), even higher than the standard compounds used (gallic acid, rutin, quercetin, and trolox) and moderate in stems and stalks. Rahman et al. [11] also proved the high activity of the leaf extracts, where the free radical scavenging activity was the highest for the ethyl acetate fraction (IC_50_ = 16.45 mg/mL) in comparison with the other subfractions (hexane, CHCI_3_, BuOH), higher than synthetic antioxidant used as a control in the study. The AOA data of the leaf extracts in our study were higher than those reported by Lungu et al. [13]. The same authors defined the observed antioxidant activity for the ethanol leaf extracts as very good. The above-mentioned authors attributed the strong antioxidant activity to the high levels of total polyphenols and flavonoids in the composition of the extracts. According to Albouchi et al. [9], the methanol extracts of the leaves and their hydro-distilled residues exhibited strong concentration-dependent antioxidant activities. The same authors proved that of the four methods used, the best is FRAP activity, followed by DPPH and ABTS. Our data also confirmed the highest activity of the leaf extracts established by the FRAP method, but the obtained values are many times higher. Other authors also reported the highest value in the FRAP test for AOA of the leaf extracts, followed by ABTS and DPPH, which regularity was also confirmed by our research [27]. According to the same team, the type of extractant and the condition of the plant parts (fresh, dried, whole, or crushed) were of essential importance for the strength of the measured activities. Probably this, as well as the differences in the method of obtaining the extracts, is the reason for the observed quantitative differences with our data. By applying ABTS and DPPH tests, Tanasković et al. [14] also demonstrated the antioxidant power of 70% bark and leaves ethanol extracts, with the higher one reported for stem barks, in contrast to the data in the present study. The authors reported the highest activity measured by the ABTS method. The main difference compared to our data is the AOA of leaves defined by FRAP method, where the highest activity was measured, higher even than flowers and bark. Other authors also demonstrated high antioxidant activity for aqueous extract (from bark and wood) with established IC_50_ of 122 ± 0.7; 201 ± 1.3, and 140 ± 0.9 μg/mL, for DPPH, ABTS, and iron reducing power test, respectively [30]. A comparison with their data could not be made due to the different quantitative reports. Other studies have found antioxidant potential for extracts derived from the fruits [38] and from the roots [8] of the tree species, as well as for individual isolated compounds included in their composition. A comparison of the AOA of the *A. altissima* flowers with data from the literature has not been made, due to the lack of such to date. The established data on the high AOA of the samples analyzed by us can definitely be related to the measured amounts of flavonoids and phenolic acids in them. The highest antioxidant activity of flower extract determined by ABTS, DPPH and CUPRAC assays could be explained with the highest established amount of rutin, quercetin, protocatechuic, chlorogenic, caffeic, syringic, *p*-coumaric and ferulic acids (Table 1).

The information in the scientific literature on the applied in vitro DNA test is also scarce. Studies by Todorova et al. [15] reported the DNA protective potential (against zeocin-induced oxidative damage) of methanol and hexane extracts obtained from the stem barks of *A. altissima*. The same authors found that the tested extracts did not have a genotoxic, mutagenic, or carcinogenic effect on model yeasts *Saccharomyces cerevisiae.* The comparative analysis between the activity of the two types of tested extracts shows a higher degree of protection with the methanol extract and a lower one with the hexane extract. The hydrophilic extractant they used (70% methanol) turned out to be better than the lipophilic one in extracting the bioactive components responsible for the observed effect. The extractant used by us (96% ethanol) has similar chemical characteristics, which would explain the similarity in the observed results for the barks, but the cited authors used much higher concentrations (10–1000 µg/mL). There are also differences regarding the concentration and type of solvent used, as well as the condition of the plant samples. The authors indicated that only the lowest applied concentration (10 µg/mL) of the methanol extract could not protect DNA from damage, whereas in our research the extracts in the higher applied concentrations (5.25–10 µg/mL) completely showed the protection of the plasmid DNA. Our data confirm the established DNA protective potential of *A. altissima* bark extracts, as well as complement the information on this effect established for flowers and leaves, which is currently missing.

In individual publications, microscopic characters on parts of *A. altissima* were studied. Bashir et al. [39] make a pharmacognostic analysis, which includes a microscopic characterization of the powder of the leaf, which consists only in the enumeration of the following microscopic characters: epidermal cells, paracytic type stomata, palisade cells, covering trichomes and oxalate crystals. Our analysis is more complete, indicates more microscopic features, and does not reveal a paracytic stomata. The findings of Abdel-Baky et al. [40] also indicate an anomocytic type of stomata by observing the leaf epidermis of *A. altissima*. These authors make a microscopic analysis of sections (not powder) of the leaf, stem, and stem bark of the ailanthus. Raja et al. [41] point out the main diagnostic features of ailanthus stem and root bark powder. Our analysis confirms the indicated microscopic features, such as sclereids, fibers, and cork, but they do not indicate another important diagnostic feature, such as oxalate crystals, which are represented in the stem bark in the form of cubes and druses.

## 4. Materials and Methods

### 4.1. Chemicals and Reagents

*HPLC analysis and antioxidant activities*. The reagents for the analyses were supplied by Sigma-Aldrich Chemie GmbH (Steinheim am Albuch, Germany): HPLC-grade solvents (acetic acid, acetonitrile, ethanol, and methanol); flavonoids—kaempherol, rutin, (−)-epicatechin, hesperidin, (+)-catechin; phenolic acids—gallic acid, vanillic acid, salicylic acid, syringic acid, chlorogenic acid, ferulic acid, protocatehuic acid, caffeic acid, *p*-coumaric acid, rosmarinic acid; potassium persulfate, sodium acetate anhydrous, DPPH (2,2-diphenyl-1-picrylhydrazyl), ABTS (2,2′-azino-bis(3-(6-hydroxy-2,5,7,8-tetramethylchroman-2-carboxylic acid), TPTZ (2,4,6-tri (2-pyridyl)1,3,5-triazine), iron (III) chloride, neocuproine, copper (II) chloride, and ammonium acetate. Trolox (6-hydroxy-2,5,7,8-tetramethylchroman-2-carboxylic acid, Merck) and methanol (Merck, Darmstadt, Germany) were used to prepare the calibration curve for antioxidant activity.

*DNA nicking protection assay.* The following reagents were used: TBE buffer—Duchefa (Haarlem, The Netherlands), 96% ethanol extra pure Ph. Eur. (Karl Roth, Karlsruhe, Germany), agarose SPI (Duchefa), Trolox (Merck), iron(II) sulfate heptahydrate, potassium phosphate dibasic (Sigma-Aldrich); di-Potassium hydrogen phosphate (Merck, Darmstadt, Germany), hydrogen peroxide solution (Sigma-Aldrich) and Whatman filter paper No. 1 (Sigma-Aldrich, Germany).

*Light-microscopic analysis*. Chloral hydrate ≥ 98.5, Ph. Eur. (Karl Roth, Karlsruhe, Germany).

### 4.2. Plant Material Collection and Preparation of the Plant Extracts

*Ailanthus altissima* aerial plant substances (flowers, leaves, and stem bark) were collected in May–June 2021 from Bulgaria, Plovdiv city (N 42°15′92.69″, E 24°73′69.55″), and they were identified at the Department of Botany, University of Plovdiv “Paisii Hilendarski”. The voucher specimen of the tree species № 063263 was deposited in an herbarium (SOA) at the Agricultural University of Plovdiv, Bulgaria.

Dried and ground plant samples used for HPLC analysis and antioxidant activity, were triplicate extracted with 70% water-ethanol (*v*/*v*), at 70 °C in a water bath (15 min) heated under reflux (hydromodule 1:10). The resulting three filtrates after filtration through filter paper and removal of the residue were used for the analyses.

Plant substances (crushed, fresh) used for the DNA analysis were extracted with 96% ethanol for 10 days. The methodology and apparatus used to prepare the dried (vacuum) extracts are according to the previously described procedure of Andonova et al. [42].

### 4.3. HPLC Analysis for Determination of the Phenolic Profile

HPLC analysis was conducted according to the method described by Krasteva and Andonova et al. [42,43] using HPLC system Waters 1525 (Binary HPLC pump) and UV-VIS Waters 2487 (Dual Absorbance Detector). Separation of the compounds was performed by a SUPELCO Analytical Discovery HS C18 column (25 cm × 4.6 mm, 5 µm) under gradient conditions with a mobile phase consisting of 1% (*v*/*v*) acetic acid in water (Mobile phase A) and methanol (Mobile phase B) at a flow rate of 1 mL per minute. The elution program was: 1–36 min 90% A and 10% B, 36–37 min 78% A and 22% B, 37–47 min—70% A and 30% B, 47–58 min 60% A and 40% B, 58–59 min 54% A and 46% B, 59–71 min 40% A and 60% B, 71–72 min—20% A and 80% B, and 72–75 min—90% A and 10% B. The detection was carried out at λ = 280 nm for gallic acid, protocatechuic acid, (+)-catechin, vanillic acid, syringic acid, (+)-epicatechin, *p*-coumaric acid, salicylic acid, hesperidin and at λ = 360 nm for rosmarinic acid, chlorogenic acid, caffeic acid, ferulic acid, rutin, quercetin, and kaempferol. The injected volume of the sample was 20 µL.

### 4.4. Methods for Determining Antioxidant Activity

#### 4.4.1. ABTS Method

The radical scavenging potential of the extracts against ABTS (2,2′-azino-bis (3-ethylbenzothiazoline-6-sulfonic acid)) was determined according to Thaipong et al. and Ivanov et al. [44,45]. ABTS radical cation (ABTS•+) was generated by mixing aliquot parts of 7.0 mmol 2,2′-azinobis (3)-ethylbenzthiazoline-6-sulfonic acid (ABTS, Sigma) in distilled water and 2.45 mmol potassium persulfate (Merck) in distilled water for 16 h at ambient temperature in darkness. Before analysis, the generated ABTS+ solution was diluted with methanol in order to obtain the final absorbance of the working solution of about 1.0 ÷ 1.1 at 734 nm. For the assay, each sample (0.15 mL) was mixed with 2.85 mL of this ABTS+ solution. After incubation for 15 min at 37 °C in darkness, the absorbance was measured at 734 nm against methanol.

#### 4.4.2. DPPH Method

DPPH (2,2-diphenil-1-picrylhydrazyl) radical scavenging activity was defined according to the procedure of Kivrak et al. and Ivanov et al. [45,46]. Each extract (0.15 mL) was mixed with 2.85 mL of freshly prepared DPPH solution (0.1 mmol in methanol). The light absorption was measured against methanol at 517 nm after 15 min incubation at 37 °C in darkness.

#### 4.4.3. CUPRAC Method

The CUPRAC (Cupric Reducing Antioxidant Capacity) assay was carried out according to the procedure of Apak et al. and Ivanov et al. [45,47]. In total, 1 mL of the 10 mmol CuCl_2_ solution was mixed with 1 mL of 7.5 mmol neocuproine (Sigma) in methanol, 1.0 mL 0.1 M ammonium acetate buffer (pH 7.0), 0.1 mL of analyzed extract, and 1.0 mL distilled water. The absorbance of the sample against a reagent blank was measured at 450 nm after incubation at 50 °C in darkness for 20 min.

#### 4.4.4. FRAP Method

The FRAP (Ferric Reducing Antioxidant Power) assay was performed according to the method of Benzie and Strain, and Ivanov et al. [45,48]. The FRAP reagent was freshly pre-pared before analysis by mixing 10 parts 0.3 M acetate buffer (pH 3.6), 1 part 10 mmol 2,4,6-tripyridyl-s-triazine (TPTZ, Fluka, Buchs, Switzerland) in 40 mmol HCl (Merck), and 1 part 20 mmol FeCl_3_·6H_2_O (Merck) in distilled water. The reaction started by mixing 3.0 mL FRAP reagent with 0.1 mL of the tested extract. After incubation for 10 min at 37 °C in darkness, the absorbance of the sample was recorded at 593 nm against a blank sample that contained 70% ethanol instead of extract.

The antioxidant activity determined by ABTS, DPPH, CUPRAC, and FRAP assays was expressed as mmol Trolox equivalents (TE) per g dry weight (DW) by using a cali-bration curve built in the range of 0.05–0.5 mmol Trolox (6-hydroxy-2,5,7,8-tetramethylchroman-2-carboxylic acid), dissolved in methanol.

### 4.5. DNA Nicking Protection Assay

The DNA protective effect of the extracts was assessed using pUC19 plasmid DNA purified from *E. coli* strain Neb10 according to Andonova et al. and Rajiv et al. [42,49]. Serial dilutions of plant extracts, starting from 0.6 μg/mL up to 10.0 μg/mL, were tested. As positive, respectively negative controls different concentrations (25, 50, and 100 μg/mL) of 6-Hydroxy-2,5,7,8-tetramethylchromane-2-carboxylic acid (Trolox, Sigma) and water were used. The reactions were incubated at 37 °C for 30 min and loaded on 1.5% agarose gel electrophoresis in 0.5× TBE buffer at 50 V for 2 h. The degree of DNA nicking was analyzed using the Gel Doc™ EZ Imaging system (Bio-Rad, Hercules, CA, USA). In build software, Image Lab Software (Biorad), was used for relative quantification of the bands’ intensity.

### 4.6. Light-Microscopic Analysis of Powdered Plant Substances

A study was conducted to establish basic diagnostic pharmacognostic features of the analyzed plant substances according to the European Pharmacopoeia [50]. The powdered plant parts were sifted through a pharmacopoeial sieve with a pore size of 0.4 mm. Immediately before microscopy, the samples were treated with a chloral hydrate solution (on heating). Microscopy was performed using a Magnum T CETI trinocular light microscope (Medline Scientific, Oxfordshire, UK) and photomicrographs were captured with a digital camera (Si 5000 5 Mpx).

### 4.7. Statistical Analysis

Sample measurements were performed in triplicate. Results are presented as means with corresponding standard deviations (±SD). Significant inter-group differences at a 99% confidence level (*p* < 0.01) were determined by one-way analysis of variance (ANOVA) followed by a post hoc Tukey HSD (Honestly Significant Difference) test (online web calculator Astatsa [51]).

## 5. Conclusions

In conclusion, the phenolic profile of *A. altissima* aerial substances showed that they are rich in antioxidants, among which the flavonoids rutin (in flowers), hesperidin (in leaves), (+)-catechin (in bark), and the phenolic acids rosmarinic and salicylic (in leaves and flowers) were the best represented from all sixteen identified phenolics. The obtained results enriched the available information about the leaves and bring a new one about the flowers and barks. In addition, rosmarinic acid in the chemical composition of *A. altissima* has not been indicated so far. The established composition could explain the demonstrated high antioxidant activity of tested ethanolic extracts, which was most strongly expressed for the flower (first demonstrated), proven by ABTS, DPPH, CUPRAC, and FRAP assays. The present study reported the in vitro DNA protection potential of *A. altissima* leaf and flower extracts for the first time. The microscopic features of the powdered flower substance of *A. altissima* were not reported until now. The obtained results for stem barks and leaves enrich the available information. The obtained results encourage further research on this invasive species, which could be a natural source of antioxidants and DNA protection.

## Figures and Tables

**Table 1:** Content of flavonoids and phenolic acids in *Ailanthus altissima* aerial plant substances (mg/g dw).

№	Compounds	Plant Substabces		
		Leaves	Stem Bark	Flowers
Flavonoids				
1	Rutin	1.02 ± 0.026 ^b^	n.d.	5.68 ± 0.142 ^a^
2	Hesperidin	2.67 ± 0.067 ^a^	n.d.	0.72 ± 0.018 ^b^
3	Quercetin	0.19 ± 0.005 ^b^	0.01 ± 0.001 ^c^	0.33 ± 0.008 ^a^
4	Kaempferol	n.d.	0.11 ± 0.003	n.d.
5	(+)-Catechin	0.95 ± 0.024 ^c^	2.15 ± 0.054 ^a^	1.55 ± 0.039 ^b^
6	(−)-Epicatechin	0.23 ± 0.006 ^b^	0.54 ± 0.014 ^a^	0.13 ± 0.003 ^c^
Phenolic acids				
7	Gallic	0.30 ± 0.008 ^a^	0.01 ± 0.001 ^c^	0.16 ± 0.004 ^b^
8	Protocatechuic	n.d.	n.d.	0.15 ± 0.004
9	Chlorogenic	0.97 ± 0.024 ^b^	0.27 ± 0.007 ^c^	1.40 ± 0.035 ^a^
10	Vanillic	0.09 ± 0.002 ^c^	0.73 ± 0.018 ^a^	0.16 ± 0.004 ^b^
11	Caffeic	0.13 ± 0.003 ^b^	n.d.	0.23 ± 0.006 ^a^
12	Syringic	0.20 ± 0.005 ^b^	0.08 ± 0.002 ^c^	0.39 ± 0.010 ^a^
13	*p*-Coumaric	0.24 ± 0.006 ^b^	n.d.	1.35 ± 0.034 ^a^
14	Ferulic	0.11 ± 0.003 ^b^	0.07 ± 0.002 ^b^	1.31 ± 0.033 ^a^
15	Salicilylic	6.19 ± 0.155 ^a^	0.07 ± 0.002 ^c^	3.81 ± 0.095 ^b^
16	Rosmarinic	10.32 ± 0.258 ^a^	0.13 ± 0.003 ^c^	2.01 ± 0.050 ^b^

n.d.—not detected; Results are presented as means with corresponding standard deviations (±SD). Different letters indicate significant differences according to Tukey’s test (*p* < 0.01).

**Table 2:** In vitro antioxidant activities of ethanolic extracts from *Ailanthus altissima* aerial plant substances.

Plant Extract	ABTS-Assay, mmol TE/g dw ^1^	DPPH-Assay, mmol TE/g dw	FRAP-Assay, mmol TE/g dw	CUPRAC-Assay, mmol TE/g dw
Leaf	299.54 ± 4.29 ^b^	225.62 ± 3.36 ^b^	906.01 ± 1.53 ^a^	548.07 ± 1.54 ^b^
Flower	893.14 ± 1.54 ^a^	729.72 ± 2.04 ^a^	661.48 ± 1.50 ^b^	789.54 ± 2.19 ^a^
Stem bark	31.24 ± 1.01 ^c^	24.96 ± 1.52 ^c^	16.65 ± 1.02 ^c^	10.22 ± 0.53 ^c^

The samples were analyzed in triplicate, and results were expressed in mean ± standard deviation (SD). Different superscript letters indicate significant differences according to Tukey’s test (*p* < 0.01). ^1^ Trolox equivalent per gram of dry weight.

## Data Availability

Not applicable.

## Supplementary Materials

The following supporting information can be downloaded at: https://www.mdpi.com/article/10.3390/plants12040920/s1, Figure S1: HPLC-chromatograms of phenolic compounds and their standards determined at 280 nm (a,b) and 360 nm (c,d) from Ailanthus altissima stem bark extracts.
