# Supplementary material for: Phenolic Profile, Antioxidant and DNA-Protective Capacity, and Microscopic Characters of Ailanthus altissima Aerial Substances
Source: Plants (Basel). 2023 Feb 17;12(4):920. doi: 10.3390/plants12040920 (PMC9967504; doi:10.3390/plants12040920)
Supplement: Supplementary file 1 [file plants-12-00920-s001.zip › plants-2219462-supplementary.pdf]

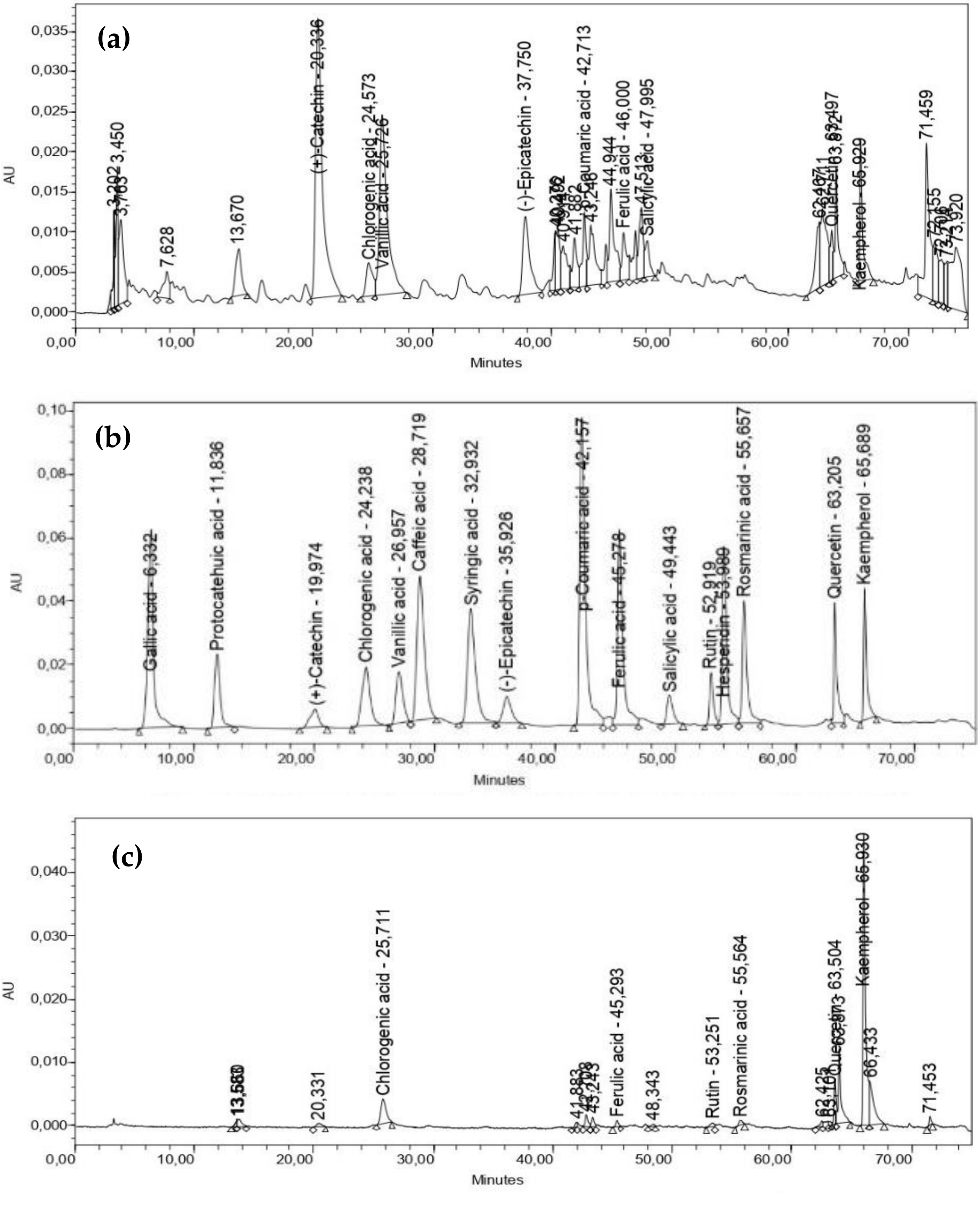

(a)
AU
Minutes
3,450
7,628
13,670
(+)-Catechin - 20,336
Chlorogenic acid - 24,573
Vanillic acid - 25,726
(-)-Epicatechin - 37,750
p-Coumaric acid - 42,713
44,944
Ferulic acid - 46,000
Salicylic acid - 47,995
Quercetin
Kaempherol - 65,929
71,459
73,920
(b)
Gallic acid - 6,332
Protocatehuic acid - 11,836
(+)-Catechin - 19,974
Chlorogenic acid - 24,238
Vanillic acid - 26,957
Caffeic acid - 28,719
Syringic acid - 32,932
(-)-Epicatechin - 35,926
p-Coumaric acid - 42,157
Ferulic acid - 45,278
Salicylic acid - 49,443
Rutin - 52,919
Hesperidin - 53,980
Rosmarinic acid - 55,657
Quercetin - 63,205
Kaempherol - 65,689
(c)
20,331
Chlorogenic acid - 25,711
41,883
43,243
Ferulic acid - 45,293
48,343
Rutin - 53,251
Rosmarinic acid - 55,564
63,425
Quercetin - 63,504
Kaempherol - 65,930
66,433
71,453

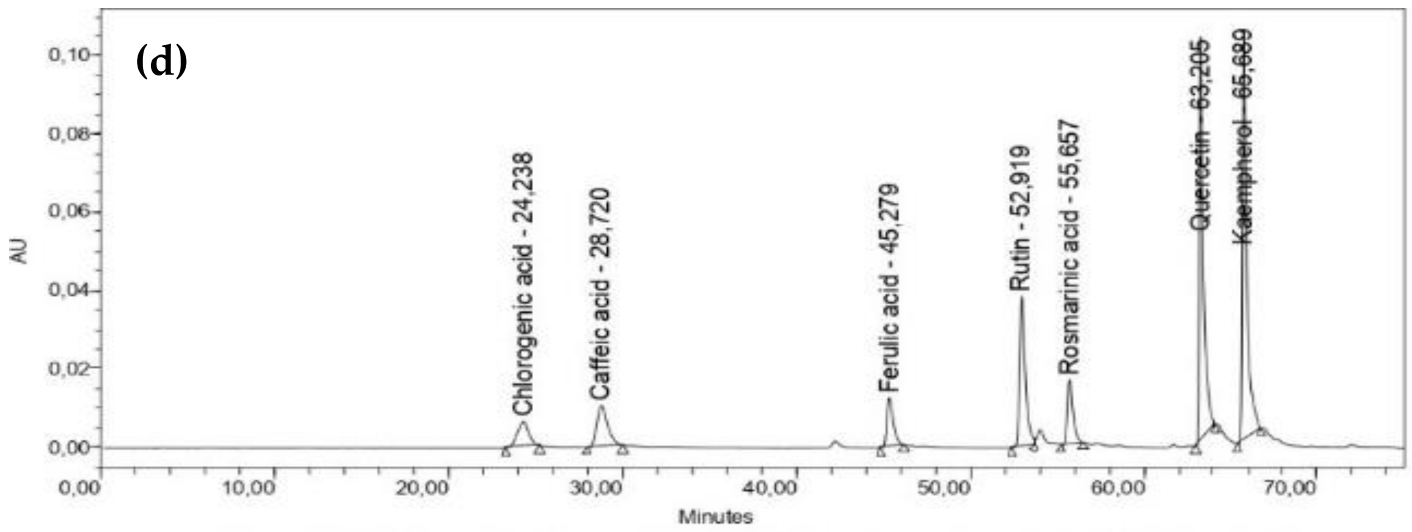

Figure S1. HPLC-chromatograms of phenolic compounds and their standards determined at 280 nm (a, b) and 360 nm (c, d) from *Ailanthus altissima* stem bark extracts.
